# Influence of alpha‐synuclein on glucose metabolism in Alzheimer's disease continuum: Analyses of α‐synuclein seed amplification assay and FDG‐PET

**DOI:** 10.1002/alz.14571

**Published:** 2025-02-12

**Authors:** Elijah Mak, Scott A. Przybelski, Heather J. Wiste, Angela J. Fought, Christopher G. Schwarz, Matthew L. Senjem, Clifford R. Jack, Val J. Lowe, Ronald C. Petersen, Bradley F. Boeve, John T. O'Brien, Kejal Kantarci, for the Alzheimer's Disease Neuroimaging Initiative

**Affiliations:** ^1^ Department of Radiology Mayo Clinic Rochester Minnesota USA; ^2^ Department of Psychiatry University of Cambridge School of Clinical Medicine Cambridge UK; ^3^ Department of Quantitative Health Sciences Mayo Clinic Rochester Minnesota USA; ^4^ Department of Information Technology Mayo Clinic Rochester Minnesota USA; ^5^ Department of Neurology Mayo Clinic Rochester Minnesota USA

**Keywords:** alpha‐synuclein, Alzheimer's disease, amyloid beta, biomarkers, brain metabolism, cognitive impairment, fluorodeoxyglucose positron emission tomography, Lewy bodies, neurodegeneration, seed amplification assay, tau

## Abstract

**INTRODUCTION:**

We investigated the association between alpha‐synuclein (α‐syn) pathology and brain glucose metabolism across the cognitive spectrum of Alzheimer's disease (AD) co‐pathologies.

**METHODS:**

Fluorodeoxyglucose positron emission tomography (FDG‐PET) data from 829 Alzheimer's Disease Neuroimaging Initiative participants (648 cognitively impaired [CI], 181 unimpaired [CU]) were compared between α‐syn seed amplification assay (SAA) positive and negative groups. Interactions with cerebrospinal fluid (CSF) AD biomarkers were examined.

**RESULTS:**

SAA+ was associated with widespread hypometabolism among CI individuals, particularly in posterior cortical regions, independent of CSF amyloid and tau levels in the occipital lobes. Regional hypometabolism mediated the effect of α‐syn SAA on disease severity in CI individuals, independent of CSF amyloid and tau levels. There were no influences of SAA on FDG‐PET in CU individuals.

**DISCUSSION:**

This study supports a model in which α‐syn aggregation influences metabolic dysfunction, which then influences clinical disease severity, independent of AD. SAA+ could help optimize participant selection and outcome measures for clinical trials in AD.

**Highlights:**

- α‐synuclein seed amplification positivity (SAA+) was associated with hypometabolism in cognitively impaired individuals.
- Hypometabolism mediated the influence of α‐synuclein on disease severity.
- Occipital hypometabolism in SAA+ was independent of cerebrospinal fluid levels of Alzheimer's disease pathology.
- These findings can optimize future clinical trials targeting α‐synuclein pathology.

## 1 BACKGROUND

The amyloid cascade hypothesis has been the dominant framework for understanding Alzheimer's disease (AD) pathogenesis, positing that the accumulation of amyloid beta (Aβ) initiates a cascade of events leading to tau hyperphosphorylation, neuroinflammation, and neurodegeneration.^1^ However, the role of co‐pathologies in AD, particularly alpha‐synuclein (α‐syn) aggregates typically associated with Parkinson's disease and Lewy body dementia, has gained increasing attention. Up to 50% of AD patients exhibit α‐syn pathology alongside classical AD hallmarks, potentially contributing to disease heterogeneity.^2^, ^3^ Moreover, recent evidence suggests that Aβ, tau, and α‐syn may interact and influence each other's propagation and co‐seeding,^4^, ^5^ forming a pathological triumvirate that exacerbates the neurodegenerative process and accelerates cognitive decline.^6^

Studying α‐syn pathology in AD has been challenging due to the lack of validated biomarkers. Unlike amyloid and tau, which can be visualized using positron emission tomography (PET) imaging, there are no approved ligands for α‐syn.^7^, ^8^ Total α‐syn levels in cerebrospinal fluid (CSF) lack specificity for pathological forms and show inconsistent results across studies.^9^, ^10^ Recent advances, leveraging experimental evidence suggesting a prion‐like propagation of α‐syn, have led to the development of an in vitro seed amplification assay (SAA) that can detect misfolded α‐syn in CSF with high sensitivity and specificity,^11^, ^12^, ^13^, ^14^ thereby allowing us to delineate the in vivo interactions of α‐syn pathology with other pathological hallmarks and their cumulative impact on neurodegenerative changes that characterize AD.

Studies using SAA have begun to reveal insights into the influences of α‐syn pathology on cognitive function in AD.^15^, ^16^ SAA positivity has been associated with detrimental effects on multiple cognitive domains, independent of other known risk factors. The co‐occurrence of α‐syn pathology with AD seems to exacerbate cognitive decline, aligning with neuropathological studies showing that the presence of Lewy bodies in AD is associated with a more severe disease course, accelerated brain atrophy,^17^ and earlier mortality.^18^, ^19^

A crucial aspect yet to be fully elucidated is how α‐syn pathology fits into the model of AD progression and its association with early neurodegenerative changes, such as synaptic dysfunction.^20^, ^21^ To this end, fluorodeoxyglucose (FDG) PET is one of the most well‐established proxies for neuronal function.^22^, ^23^ While FDG PET studies have characterized distinct topographic hypometabolism patterns in AD and Lewy body diseases as early as the prodromal stages,^24^, ^25^, ^26^, ^27^, ^28^, ^29^, ^30^ specific metabolic signatures remain unknown. Recently, Collij et al. demonstrated that α‐syn pathology detected by SAA exacerbates AD progression through accelerated cognitive decline.^31^ Nevertheless, the precise role of metabolic dysfunction in mediating the relationships between α‐syn pathology and clinical manifestations—including both clinical disease severity and domain‐specific cognitive impairment—remains to be elucidated. Understanding these mechanistic relationships could have important implications for patient stratification and development of disease‐modifying therapies in AD.

Using data from the Alzheimer's Disease Neuroimaging Initiative (ADNI), we aimed to delineate the relationship between α‐syn pathology and regional brain glucose metabolism across the AD continuum. We used the clinically validated Amprion SAA to quantify α‐syn pathology in CSF samples from a large cohort spanning cognitively unimpaired (CU) individuals to mild cognitive impairment (MCI) and those with mild‐to‐moderate AD dementia. Our primary objectives were to (1) investigate the association between α‐syn pathology and regional brain glucose metabolism and the extent to which it is independent of apolipoprotein E (*APOE*) status and AD pathology, (2) examine the interactions between SAA positivity and established biomarkers of AD on FDG PET metabolism, and (3) determine whether hypometabolism might serve as a mechanistic link between α‐syn pathology and cognitive impairment within the AD spectrum.

## 2 METHODS

### 2.1 Study design and participants

This study used data from the ADNI database (https://adni.loni.usc.edu/). The study population included CU individuals, individuals with MCI due to AD, and those with a clinical diagnosis of dementia due to AD from the ADNI database. Enrollment criteria for CU participants included a Mini‐Mental State Examination (MMSE) score of 24 to 30; a Clinical Dementia Rating (CDR) of 0; and absence of depression, MCI, or dementia. MCI participants were required to have MMSE scores of 24 to 30; subjective memory complaints; objective memory loss (adjusted for education) on the Wechsler Memory Scale Logical Memory II; a CDR of 0.5; no significant impairment in other cognitive domains, essentially preserved activities of daily living; and no dementia. Participants with dementia due to AD met the National Institute of Neurological and Communicative Disorders and Stroke/Alzheimer's Disease and Related Disorders Association criteria for probable AD and a CDR of 0.5 or 1.0. Exclusion criteria at ADNI enrollment included significant neurological diseases other than AD; contraindications to neuroimaging or other ADNI protocols; neuroimaging evidence of infection, infarction, lacunes, or other focal lesions; psychiatric disorders (including psychotic features); alcohol abuse; significant systemic illness or unstable medical conditions; laboratory abnormalities that could complicate the study; use of specific psychoactive medications; and participation in other clinical trials. The ADNI study was conducted in accordance with the Declaration of Helsinki, and the procedures were approved by the institutional review boards of all participating sites. All participants provided written informed consent at enrollment.

### 2.2 CSF α‐syn SAA processing

CSF samples were initially collected into tubes supplied to each participating ADNI site, transferred to polypropylene transfer tubes, and then frozen on dry ice within 1 hour of collection. These samples were shipped overnight on dry ice to the ADNI Biomarker Core laboratory at the University of Pennsylvania Medical Center. Upon arrival at the ADNI Biomarker Core laboratory, the CSF samples were thawed and aliquoted into 0.5 mL cryo for long‐term storage at −80°C. Pristine CSF aliquots were provided for SAA analysis. The α‐syn SAA testing was performed by Amprion Clinical Laboratory (CLIA ID No. 05D2209417; CAP No. 8168002) using a clinically validated method in compliance with Clinical Laboratory Improvement Amendment (CLIA) standards. The analysis protocol involved testing each CSF sample in triplicate within a 96‐well plate. The reaction mixture, totaling 100 µL, consisted of 100 mM PIPES (pH 6.5), 0.44 M NaCl, 0.1% sarkosyl, 10 µM Thioflavin T (ThT), 0.3 mg/mL recombinant α‐syn, and 40 µL CSF.

**RESEARCH IN CONTEXT**

1. **Systematic review**: The authors conducted a literature search using PubMed on the alpha‐synuclein (α‐syn) seed amplification assay (SAA) and its relationship with brain metabolism measured by fluorodeoxyglucose positron emission tomography. While recent work has demonstrated that α‐syn pathology detected by SAA exacerbates Alzheimer's disease (AD) progression through accelerated cognitive decline, no studies have specifically investigated the mechanistic pathway linking SAA positivity to cognitive impairment through brain metabolism.
2. **Interpretation**: SAA positivity was associated with hypometabolism in cognitively impaired individuals, which was independent of cerebrospinal fluid AD pathology in the occipital lobes. Mediation analyses suggested that α‐syn pathology may contribute to cognitive impairment through its effect on brain metabolic dysfunction. These findings expand the understanding of α‐syn's role in neurodegenerative processes beyond classical synucleinopathies and provide insights into the mechanistic pathway linking α‐syn accumulation to cognitive impairment.
3. **Future directions**: Longitudinal studies should examine the temporal dynamics between α‐syn accumulation and brain metabolism, evaluate SAA as a biomarker for prodromal Lewy body dementia, and explore its associations with AD biomarkers.

Two silicon nitride beads were added to each well to enhance consistency. Positive and negative assay quality control samples were included on each plate to ensure accuracy. Plates were sealed with an optical adhesive film and placed in a BMG LABTECH FLUOstar Microplate Reader for incubation and measurement. The incubation was conducted at 42°C for 20 hours, with cycles consisting of 1 minute of shaking followed by 14 minutes of rest. Fluorescence measurements were recorded after each shake using an excitation wavelength of 440 nm and an emission wavelength of 490 nm. After 20 hours, the maximum fluorescence intensity for each well was logged. An algorithm was then applied to the triplicate readings for result categorization.

CSF samples were classified into one of four categories: “PD/DLB‐like Detected” (Type 1) if α‐syn aggregates were consistent with seeds observed in Parkinson's disease and dementia with Lewy bodies (DLB); “MSA‐like Detected” (Type 2) if α‐syn aggregates matched seeds that are typically seen in multiple system atrophy; “Not Detected” if no α‐syn aggregates were observed; or “Indeterminate” if samples did not yield a definite result after two tests. For all subsequent analyses in this study, only Type 1 cases (*n* = 196; 34 CU and 162 CI) were considered SAA+, and only “Not Detected” cases (*n* = 633; 147 CU and 486 CI) were considered SAA–. Both Type 2 (*n* = 2; 1 CU and 1 CI) and Indeterminate cases (*n* = 7; 1 CU and 6 CI) were excluded. All CSF α‐syn SAA analyses were performed with analysts blinded to participants’ demographic details, clinical profiles, and AD biomarker data.

### 2.3 AD CSF biomarker assessments

Pristine CSF aliquots were analyzed using electrochemiluminescence immunoassays (ECLIA) on a fully automated Elecsys cobas e 601 instrument. The Roche Elecsys CSF immunoassays for Aβ42, phosphorylated tau181 (p‐tau181), and total tau were used following the Roche Study Protocol at the ADNI Biomarker Laboratory, in accordance with the kit manufacturer's instructions. A single lot of reagents was used for each measured biomarker. The analyses were conducted in a series of runs between November 17, 2016, and June 22, 2022. Each sample was analyzed once (in singlicate) for each biomarker test. A standard new lot rollover protocol from the manufacturer was followed, involving repeated analyses of quality control samples. The analyte measuring ranges (from lower to upper technical limits) for each biomarker were as follows: (1) Elecsys CSF Aβ42 immunoassay: 200 to 1700 pg/mL, and (2) Elecsys CSF p‐tau181 immunoassay: 8 to 120 pg/mL. Results outside these ranges (above the upper or below the lower technical limits) were excluded from the relevant analyses described in this study. For biomarker positivity classification, we used previously established cutoff values.^16^ Participants were classified as Aβ42 positive if their CSF Aβ42 levels were below 980 pg/mL, and p‐tau181 positive if their CSF p‐tau181 levels > 24 pg/mL. Overall AD CSF biomarker positivity was determined using the CSF p‐tau181/Aβ42 ratio, with values > 0.025 indicating an AD‐like profile.

### 2.4 FDG PET processing

FDG PET images were obtained on multiple scanners at 62 sites with protocols specific to platforms. Dynamic 3D scans of six 5 minute frames were acquired 30 to 60 minutes after injections of 185 MBq of 18F‐FDG. All original ADNI FDG PET scans underwent standardized image pre‐processing steps to improve uniformity across the scanners. Detailed information on FDG PET acquisition and pre‐processing is available on the ADNI website (http://adni.loni.usc.edu/methods/documents/). For the current study, we selected FDG PET scans acquired within 6 months of the CSF sample collection used for SAA analysis. In cases in which multiple scans were available within this timeframe, the scan closest to the CSF collection date was chosen. Regional standardized uptake value ratios (SUVRs) were extracted using the Mayo Clinic Adult Lifespan Template (MCALT) atlas, which provides a detailed parcellation of the brain into anatomically and functionally relevant regions of interest (ROIs).^32^ Given the regional susceptibility of the substantia nigra in Lewy body disease, we also included a specific segmentation of this region based on previous studies.^26^, ^33^

### 2.5 Statistical analyses

Descriptive statistics were computed for demographic, clinical, and biomarker measures associated at the time of CSF sample collection and compared between α‐syn SAA+ and SAA− groups within cognitive impairment groups using two‐sample *t* tests for continuous variables and Pearson chi‐square tests for categorical variables. The CSF biomarkers were analyzed with log transformations due to skewness. To evaluate the influence of α‐syn SAA positivity on regional brain glucose metabolism, we constructed linear regression models for each ROI separately in the cognitively impaired (CI) and CU groups. The dependent variable was the SUVR of regional FDG PET, and the independent variables included α‐syn SAA status, age, sex, *APOE* carrier status, and site. The site variable was included to adjust for potential variability from scanning across different sites, such as differences in PET and magnetic resonance imaging (MRI) scanner brand and hardware. To determine the extent to which AD pathologies drove the influences of α‐syn SAA positivity, we expanded the previous model and controlled for concomitant AD pathologies using the CSF p‐tau181/Aβ42 ratio based on recent work establishing the CSF p‐tau181/Aβ42 ratio as a robust biomarker for AD diagnosis and staging.^34^

After isolating the influences of α‐syn SAA positivity on regional metabolism by controlling for CSF p‐tau181/Aβ42, we derived a composite ROI encompassing the intersection of regions associated with the residual influences of α‐syn SAA. We then ran post hoc analyses testing for the interactions of α‐syn SAA with CSF p‐tau181/Aβ42 ratio to assess whether CSF levels of AD biomarkers moderate the effect of α‐syn SAA status on FDG PET SUVR. Finally, we implemented a mediation analysis using the R package “mediation” to evaluate whether regional FDG PET uptake mediated the association between SAA α‐syn+ status and clinical disease severity measure Clinical Dementia Rating Sum of Boxes (CDR‐SB). The mediation model specified α‐syn SAA status as the independent variable, FDG PET SUVR for each ROI as the mediator, and CDR‐SB as the dependent variable. In these models, we also controlled for age, sex, site, *APOE* carrier status, and CSF p‐tau181/Aβ42 ratio to isolate the influence of α‐syn SAA status on CDR‐SB through its association with FDG PET. For each FDG PET ROI, the following models were fitted:

- $FD\;G_{\mathrm{SUV}R_{\mathrm{ROI}}}=\beta 0+\beta 1\;*\;Age+\beta 2\;*\;\mathrm{Sex}\;+\beta 3\;*\;\mathrm{Site}\;+\beta 4\;*APOE\;\mathrm{carrier}\;\mathrm{status}+\beta 5\;*\mathrm{CSF}\;P-Tau\;181\;/A\beta 42\;\mathrm{ratio}+\beta 6\;*\mathrm{SA}A_{\mathrm{status}}+\varepsilon 1$
- $\mathrm{CDR}-\mathrm{SB}\;=\gamma 0+\gamma 1\;*\;Age+\gamma 2\;*\;\mathrm{Sex}\;+\gamma 3\;*\;\mathrm{Site}\;+\gamma 4\;*\;APOE\;\;\mathrm{carrier}\;\mathrm{status}+\gamma 5\;*\;\mathrm{CSF}\;P-Tau\;181\;/A\beta 42+\gamma 6*\;\mathrm{SA}A_{\mathrm{status}}+\gamma 7\;*\;\mathrm{FD}G_{\mathrm{SUV}R_{\mathrm{ROI}}}+\varepsilon 2$

**FIGURE 1:**
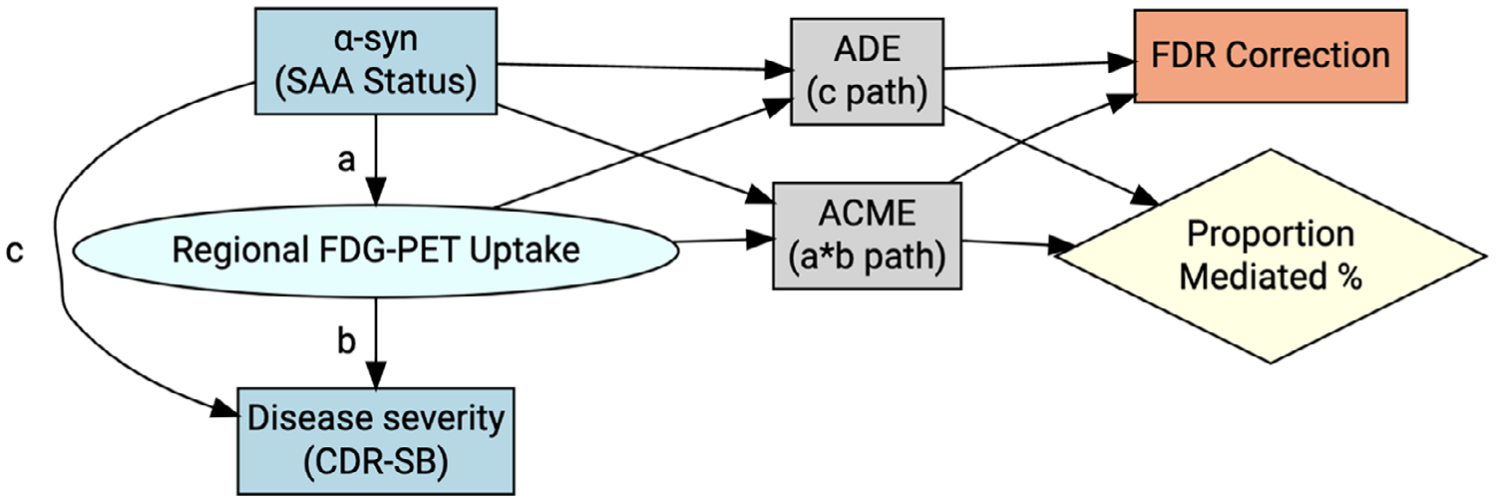
Schematic representation of the causal mediation analysis examining the relationship between α‐syn SAA status, regional brain glucose metabolism measured by FDG PET, and clinical disease severity assessed by the CDR‐SB. The diagram illustrates the direct effect (c' path) of α‐syn SAA status on cognitive performance and the indirect effect mediated through regional FDG PET uptake (ab path). The ACME represents the indirect effect of α‐syn SAA status on cognitive performance through its influence on regional brain metabolism. The ADE represents the direct effect of α‐syn SAA status on cognitive performance, independent of regional brain metabolism. Both ACME and ADE *P* values undergo FDR correction to account for multiple comparisons across ROI. α‐syn, alpha‐synuclein; ACME, average causal mediation effect; ADE, average direct effect; CDR‐SB, Clinical Dementia Rating Sum of Boxes; FDG PET, fluorodeoxyglucose positron emission tomography; FDR, false discovery rate; FDR, false discovery rate; ROI, regions of interest; SAA, seed amplification assay

The mediation function was then used to estimate the average causal mediation effect (ACME), average direct effect (ADE), and total effect. The significance of the indirect effect was tested using quasi‐Bayesian confidence intervals with 1000 iterations. False discovery rate (FDR) correction was applied to the *P* values of the ACME, ADE, and total effect for each cognitive measure to account for multiple comparisons across ROIs. The proportion of the total effect mediated by FDG PET uptake was calculated for significant mediation pathways. Results were summarized across all ROIs, highlighting those with significant mediation effects after FDR correction. All statistical analyses were performed using R. Statistical significance was set at *P* < 0.05 for demographic comparisons and FDR‐adjusted *q* < 0.05 for regional FDG PET analyses. A schematic of the mediation analysis is illustrated in Figure 1.

## 3 RESULTS

### 3.1 Sample characteristics

Detailed demographics, cognitive measures, and biomarker characteristics of the participants are presented in Table 1. Among the CU group, there were no significant differences in age, sex, proportion of *APOE* ε4 carriers, and education level between the SAA+ and SAA− groups. Within the CI group, the SAA+ group was significantly older (75.4 ± 7.3 years) than the SAA− group (73.3 ± 8.0 years, *P* = 0.003), and had a higher proportion of *APOE* ε4 carriers (62% vs. 50%, *P* = 0.008), a lower MMSE score (24.5 ± 4.1 vs. 26.4 ± 3.4, *P* < 0.001), and a higher CDR‐SB score (3.6 ± 2.5 vs. 2.5 ± 2.1, *P* < 0.001).

**TABLE 1:** Demographic, clinical, and biomarker characteristics within the unimpaired and impaired groups, stratified by CSF α‐syn SAA results at the time of CSF sample collection.

	Cognitively unimpaired			Cognitively impaired		
	SAA− *n* = 147	SAA+ *n* = 34	*p* value	SAA− *n* = 486	SAA+ *n* = 162	*p* value*
Age, years	73.9 (7.0)	75.8 (5.5)	0.14	73.3 (8.0)	75.4 (7.3)	0.003
Females, no. (%)	74 (50%)	19 (56%)	0.56	199 (41%)	62 (38%)	0.55
*APOE* ε4, no. (%)	47 (32%)	12 (35%)	0.71	242 (50%)	100 (62%)	0.008
Education, years	16.3 (2.6)	16.0 (3.2)	0.54	15.9 (2.7)	16.2 (2.8)	0.16
MMSE	29.1 (1.1)	28.7 (1.2)	0.073	26.4 (3.4)	24.5 (4.1)	<0.001
CDR‐SB	0.1 (0.2)	0.0 (0.1)	0.24	2.5 (2.1)	3.6 (2.5)	<0.001
Aβ42	1396.0 (611.5)	1280.2 (698.1)	0.20	988.9 (607.0)	764.7 (489.7)	<0.001
Abnormal Aβ 42, *n* (%)	18 (12%)	5 (15%)	0.70	213 (44%)	93 (57%)	0.003
CSF p‐tau	22.0 (9.4)	21.5 (8.6)	0.91	29.2 (16.0)	31.2 (16.0)	0.051
Abnormal p‐tau, *n* (%)	52 (35%)	10 (29%)	0.51	267 (55%)	103 (64%)	0.054
CSF p‐tau181/Aβ42 ratio	0.020 (0.017)	0.024 (0.022)	0.32	0.041 (0.033)	0.053 (0.038)	<0.001
Abnormal p‐tau181/Aβ42 ratio, no. (%)	34 (23%)	11 (32%)	0.26	290 (60%)	129 (80%)	<0.001
Dementia, no. (%)				143 (29%)	90 (56%)	<0.001

*Note*: Characteristics table of groups with the mean (SD) listed for the continuous variables and count (%) for the categorical variables.

Abbreviations: α‐syn, alpha‐synuclein; Aβ, amyloid beta; *APOE*, apolipoprotein E; CDR‐SB, clinical dementia rating sum of boxes score; CSF, cerebrospinal fluid; MMSE, Mini‐Mental State Examination; p‐tau, phosphorylated tau; SAA, seed amplification assay; SAA−, α‐syn seeding aggregates not detected; SAA+, α‐synuclein aggregates detected with an aggregation profile consistent with the characteristic seeding seen in Lewy body diseases; SD, standard deviation.

* *P* values for differences between groups were derived from *t* test for continuous variables and a chi‐squared test for categorical variables.

### 3.2 Associations of SAA positivity with FDG PET uptake

**FIGURE 2:**
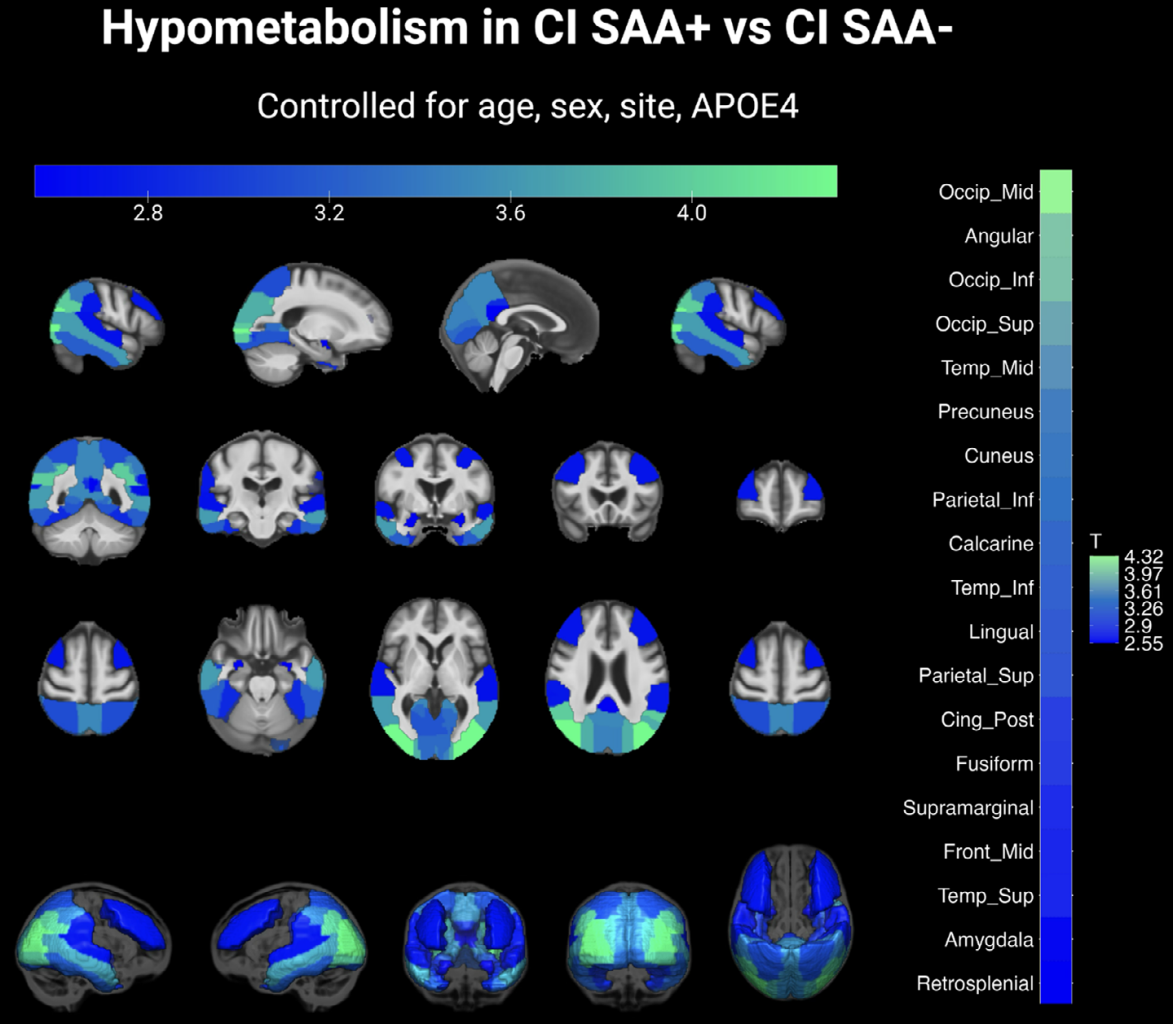
Regional FDG PET uptake comparison between α‐syn SAA+ and SAA− in CI individuals. *T*‐statistics are overlaid on the MCALT brain template, for regions where FDG uptake significantly differs between SAA+ and SAA− individuals (FDR corrected *q* < 0.05). Colors represent the *t* statistics ranging on an increasing scale from blue to green. Higher *t* statistics display a greater degree of hypometabolism in SAA+ individuals. Brain slices are shown in three orientations: sagittal (top row), coronal (middle row), and axial (bottom row). α‐syn, alpha‐synuclein; *APOE*, apolipoprotein E; CI, cognitively impaired; FDG PET, fluorodeoxyglucose positron emission tomography; FDR, false discovery rate; MCALT, Mayo Clinic adult lifespan template; SAA, seed amplification assay; SAA−, α‐syn seeding aggregates not detected; SAA+, α‐synuclein aggregates detected with an aggregation profile consistent with the characteristic seeding seen in Lewy body diseases

Regional FDG PET SUVRs were compared between α‐syn SAA+ and α‐syn SAA− in both the CI and CU groups, adjusting for potential confounds of age, sex, *APOE* ε4 carrier status, and site. In the CI group, SAA+ individuals showed significantly lower FDG uptake in 18 of the 48 ROIs analyzed (FDR *q* < 0.05). The *t* statistics for regions with significantly lower FDG uptake in SAA+ compared to SAA− individuals are displayed on the MCALT brain template in Figure 2. The strongest effects were observed in posterior cortical regions, with the middle occipital gyrus showing the most significant hypometabolism (*t* = 4.33), followed by the angular gyrus (*t* = 4.00) and inferior occipital gyrus (*t* = 3.97). Additional regions showing significant hypometabolism included the superior occipital gyrus (*t* = 3.79), middle temporal gyrus (*t* = 3.65), precuneus (*t* = 3.51), and inferior parietal cortex (*t* = 3.42), extending into other temporal and frontal regions. In the CU group, no regions showed statistically significant differences between SAA+ and SAA− individuals.

### 3.3 Associations of SAA positivity with FDG PET uptake, additionally controlling for the CSF p‐tau181/Aβ42 ratio and *APOE*

**FIGURE 3:**
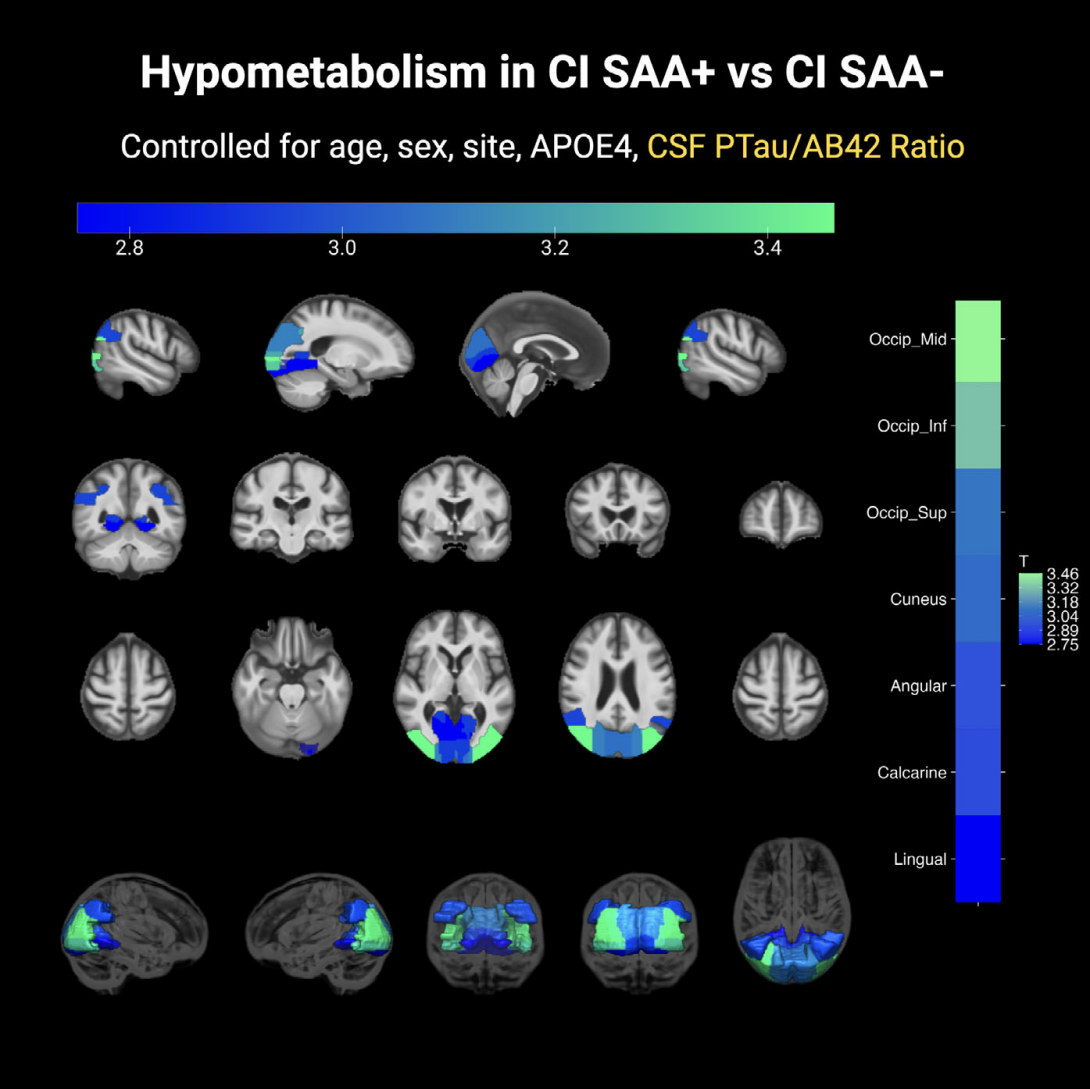
Regional FDG PET uptake comparison between α‐syn SAA+ and SAA− in CI individuals, adjusting for AD pathology using the CSF p‐tau181/Aβ42 ratio. *T* statistics are overlaid on the MCALT brain template, for regions where FDG uptake significantly differs between SAA+ and SAA− individuals (FDR corrected *q* < 0.05). Colors represent the *t* statistics ranging on an increasing scale from blue to green. Higher *t* statistics display a greater degree of hypometabolism in SAA+ individuals. Brain slices are shown in three orientations: sagittal (top row), coronal (middle row), and axial (bottom row). Aβ, amyloid beta; α‐syn, alpha‐synuclein; AD, Alzheimer's disease; *APOE*, apolipoprotein E; CI, cognitively impaired; CSF, cerebrospinal fluid; FDG PET, fluorodeoxyglucose positron emission tomography; FDR, false discovery rate; MCALT, Mayo Clinic adult lifespan template; p‐tau, phosphorylated tau; SAA, seed amplification assay; SAA−, α‐syn seeding aggregates not detected; SAA+, α‐synuclein aggregates detected with an aggregation profile consistent with the characteristic seeding seen in Lewy body diseases

In multivariable regression models comparing regional FDG PET uptake between CI SAA+ and SAA− groups in CI, we additionally controlled for the main effects of CSF AD biomarkers and *APOE* ε4 carrier status. Our analysis revealed that the CSF p‐tau181/Aβ42 ratio significantly influenced widespread FDG reductions (FDR *q* < 0.05). The *t* statistics for regions with significantly lower FDG uptake in SAA+ compared to SAA− individuals, after accounting for the CSF p‐tau181/Aβ42 ratio are displayed on the MCALT brain template in Figure 3. Notably, despite the significant contributions of CSF p‐tau181/Aβ42 ratio to widespread hypometabolism, SAA positivity was independently associated with significant hypometabolism in several regions of the visual cortex across all models, including the middle and inferior occipital cortices, calcarine, lingual gyrus, and the cuneus (FDR *q* < 0.05; Figure S1 in supporting information).

### 3.4 Interaction analysis with CSF p‐tau181/Aβ42 ratio

To further investigate the relationship between α‐syn pathology and AD biomarkers, we created a meta‐ROI from the occipital regions demonstrating significant hypometabolism in our previous analysis controlling for AD biomarkers (middle, inferior, superior occipital gyrus, cuneus, calcarine, angular and lingual gyrus). There was a trend toward significance for the interaction between SAA+ status and the CSF p‐tau/Aβ42 ratio (*β* = 0.032, standard error = 0.019, *P* = 0.096).

### 3.5 Mediation analyses

**FIGURE 4:**
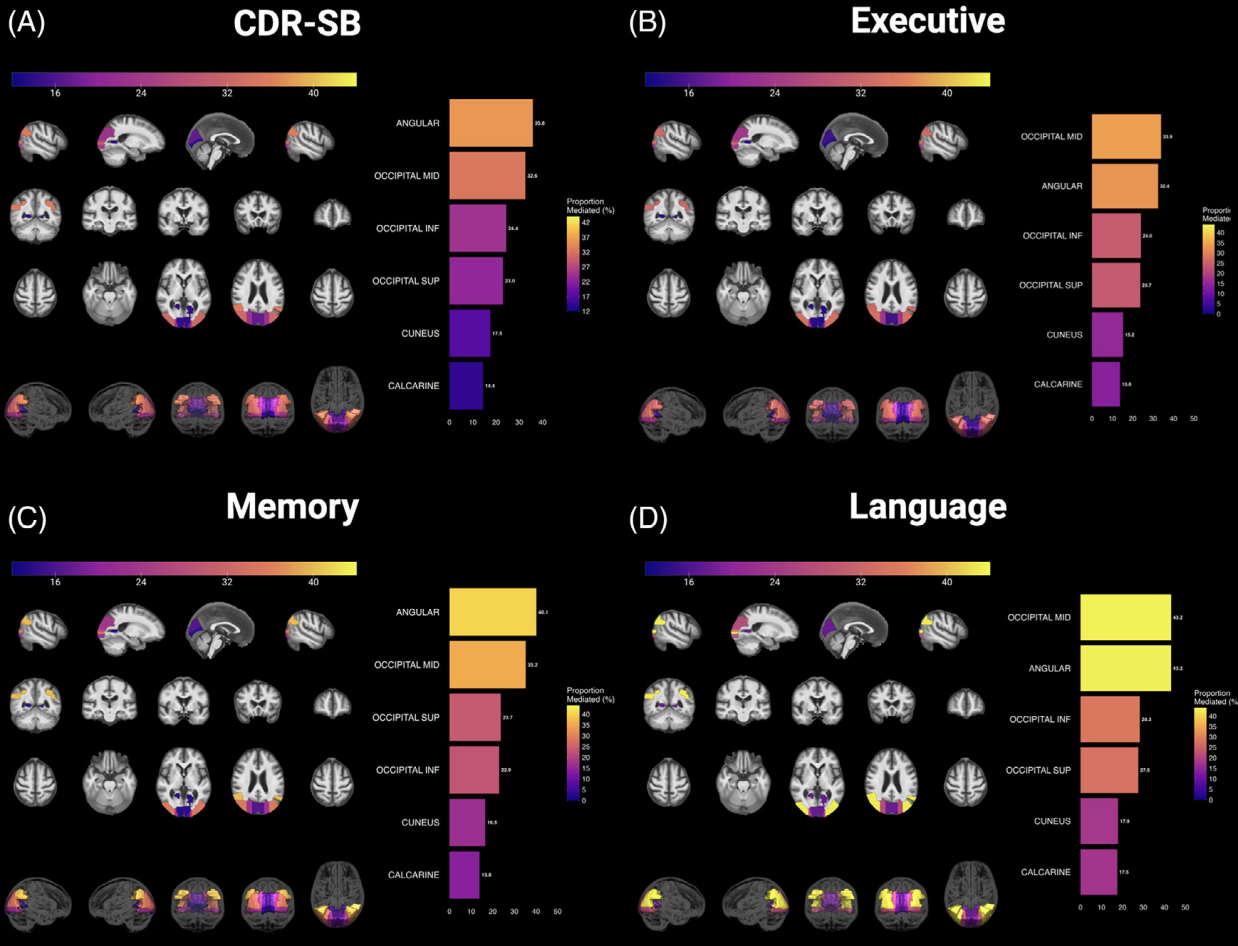
Regional mediation analyses of FDG PET on the relationship between α‐syn SAA+ and clinical disease severity, measured using the (a) CDR‐SB, and (b–d) cognitive domains in the CI group, adjusted for the age, sex, site, *APOE* carrier status, and the CSF p‐tau181/Aβ42 ratio. The color bar represents the proportion of the effect mediated (%). The images show sagittal, coronal, and axial views. The bottom row includes 3D renderings of the brain showing the distribution of the significant mediation effects (ACME FDR *q* < 0.05). Aβ, amyloid beta; α‐syn, alpha‐synuclein; ACME, average causal mediation effect; *APOE*, apolipoprotein E; CDR‐SB, Clinical Dementia Rating Sum of Boxes; CI, cognitively impaired; CSF, cerebrospinal fluid; FDG PET, fluorodeoxyglucose positron emission tomography; FDR, false discovery rate; p‐tau, phosphorylated tau; SAA, seed amplification assay; SAA+, α‐synuclein aggregates detected with an aggregation profile consistent with the characteristic seeding seen in Lewy body disease

To further understand the influence of SAA+ and hypometabolism on disease severity and cognition, we conducted mediation analyses investigating how FDG PET metabolism mediates the relationship between α‐syn and clinical or cognitive outcomes. All mediation analyses were adjusted for age, sex, site, *APOE* ε4 carrier status, and the CSF p‐tau181/Aβ42 ratio. In our analysis of CDR‐SB (our primary clinical outcome), 6 of the 48 ROIs (12.5%) showed statistically significant mediation effects (ACME indirect FDR *q* < 0.05). These regions were the angular gyrus, middle occipital gyrus, inferior occipital gyrus, superior occipital gyrus, cuneus, and calcarine cortices. Figure 4 visualizes all ROIs with significant mediation effects and their corresponding proportion of effect mediated (%). Significant ADEs were observed in all regions, indicating that α‐syn pathology also directly influences disease severity independent of its effects on brain metabolism (all ADE FDR *q* < 0.05, Figure S2 in supporting information). Analysis of domain‐specific cognitive measures across language, memory, visual, and executive function revealed a pattern of significant mediation effects that mirrored those seen with CDR‐SB (Figure 4). However, no brain regions showed significant mediation effects for visual function (all indirect FDR *q* > 0.05).

## 4 DISCUSSION

### 4.1 Summary of key findings

Our investigation into the metabolic influences of α‐syn pathology across the AD spectrum, leveraging both FDG PET and the SAA biomarker, revealed several key findings: (1) α‐syn SAA+ was associated with a posterior‐dominant topography of hypometabolism exclusively among CI individuals; (2) the metabolic signatures of α‐syn SAA+ were independent of *APOE* ε4 carrier status and AD pathology, particularly in the occipital cortex; (3) the clinical relevance of α‐syn SAA+ was corroborated through a mediation framework, which implicated hypometabolism as the mediating factor through which α‐syn exerts its detrimental influences on clinical disease severity. These findings highlight the influence of co‐existing α‐syn on synaptic dysfunction and disease severity in those with AD and non‐synucleinopathy dementia and could inform clinical trial designs in the future.

### 4.2 α‐syn SAA+ and posterior‐dominant hypometabolism in the CI group

Compared to α‐syn SAA– individuals, the SAA+ group exhibited extensive hypometabolism characterized by a posterior‐dominant topography. Interestingly, the metabolic influences of α‐syn pathology appeared to be more extensive than its impact on brain atrophy, in keeping with evidence suggesting that FDG PET is preferentially sensitive to earlier neurodegenerative events in AD compared to structural changes detected by structural MRI.^21^ A previous study showed that the impact of SAA α‐syn+ was notably confined to smaller nucleus basalis of Meynert volumes, with limited influences on widespread atrophy patterns typically observed in CI memory clinic populations.^35^ The association between α‐syn pathology and glucose hypometabolism may offer insights into the mechanisms of neurodegeneration in AD. α‐syn aggregates may impair synaptic function by disrupting vesicle trafficking and sequestering essential proteins,^36^ leading to reduced glucose metabolism as observed on FDG PET scans. Interestingly, controlling for the AD biomarker—CSF p‐tau181/Aβ42 ratio—in our models led to a notable decrease in the spatial extent of SAA+‐related hypometabolism, with residual effects primarily localized to the occipital cortices. This finding may indicate a distinct signature of α‐syn–related neurodegeneration.^25^, ^26^, ^30^, ^37^ The occipital‐dominant pattern of hypometabolism diverges from typical AD pathology and may represent a distinct signature of α‐syn–related neurodegeneration,^38^, ^39^ and raises questions about the role of α‐syn in shaping the diverse clinical presentations observed across the AD spectrum. For instance, severe occipital involvement has been linked to a higher prevalence and increased likelihood of developing visual hallucinations in DLB.^40^

### 4.3 Absence of effects of α‐syn on glucose metabolism in the CU group

The differential impact of SAA status on FDG PET between cognitive groups—with significant hypometabolism in CI SAA+ individuals but no differences in CU individuals—deserves further consideration and could be attributed to several factors. First, it could reflect a threshold effect such that while SAA can detect the presence of pathological α‐syn, the burden may need to reach a critical threshold before manifesting as detectable metabolic changes seen on FDG PET. In the CI individuals, such a putative threshold has likely been exceeded and potentially compounded by other pathological processes inherent in MCI and AD. In contrast, CU individuals, despite harboring detectable α‐syn pathology, may not yet have reached the burden necessary to disrupt glucose metabolism. Second, our smaller CU sample sizes, particularly in those with SAA+ status (*n* = 34), may have limited our ability to detect subtle metabolic differences. Nevertheless, the ability to detect α‐syn pathology before significant metabolic changes and cognitive impairment positions SAA as a promising valuable early biomarker for identifying individuals who may benefit from future disease‐modifying interventions at a stage when intervention may be most effective.

### 4.4 Hypometabolism mediates the influence α‐syn of SAA+ on cognition

While our findings align with established FDG PET literature demonstrating distinct metabolic profiles in DLB,^26^, ^40^, ^41^ the clinical implications of reduced FDG PET signal associated with α‐syn within the AD spectrum remained unclear. The present findings are therefore in support of and complement recent work by Collij et al., who demonstrated that α‐syn pathology detected by SAA is associated with both accelerated cognitive decline and posterior cortical hypometabolism.^31^ Our mediation analyses extend those findings by revealing a consistent posterior‐dominant pattern through which α‐syn impacts both global clinical severity and domain‐specific cognitive functions, effectively bridging separate lines of evidence that have previously implicated CSF α‐syn in metabolic dysfunction,^42^ cognitive decline,^15^ and disease severity.^43^

Our mediational models revealed that FDG metabolism across a core set of posterior regions—particularly the occipital areas, angular gyrus, and calcarine cortex—significantly mediated the influence of α‐syn on cognition. Critically, this pattern persisted after adjusting for *APOE* status and AD pathology. Rather than showing distinct regional patterns that mapped onto specific domains, the shared posterior‐dominant pattern of regional mediation across our models points toward a common metabolic pathway through which α‐syn affects clinical severity and cognitive impairment. This aligns with our finding of residual hypometabolism in occipital regions after controlling for CSF p‐tau/Aβ42 ratio and *APOE*, suggesting these posterior metabolic changes may be a key signature of α‐syn's influence on cognition. The identification of this consistent posterior metabolic pathway has important implications for clinical trials targeting α‐syn pathology.^44^ These regions could potentially serve as valuable biomarkers for monitoring disease progression and treatment response, particularly given their robust involvement across multiple cognitive domains.

Interestingly, we noted an absence of significant mediation effects for visual function after FDR correction despite prominent SAA‐related metabolic deficits in the posterior regions. This spatial dissociation between metabolic changes and visual processing raises intriguing possibilities about how α‐syn differentially affects the visuospatial domain. Specifically, α‐syn's impact on visual processing may involve alternative pathways such as neuroinflammation,^45^ or disruption of specific neurotransmitter systems.^46^ Future studies examining this mediation effect is present in pure DLB versus AD with co‐existing α‐syn pathology could provide valuable insights into disease‐specific mechanisms and potential therapeutic targets.

### 4.5 Strengths and limitations

The strengths of our study include large sample size, the use of Amprion's clinically validated SYNTap SAA for accurate detection of misfolded α‐syn in CSF, and a comprehensive multimodal approach integrating CSF biomarkers with FDG PET. While our mediation analysis implicitly assumes a causal pathway, longitudinal studies will be necessary to confirm causality and elucidate the temporal sequence of α‐syn aggregation, metabolic changes, and cognitive decline in individuals with AD pathology. It is also important to acknowledge the potential heterogeneity of pathologies that may be present in our sample, as autopsy studies on neuropathological findings in ADNI participants have revealed that a significant proportion of clinically diagnosed AD cases had comorbid Lewy body pathology at autopsy.^47^, ^48^ Further studies are needed to disentangle the specific contributions of AD and DLB pathologies to metabolic dysfunction and cognitive decline, potentially through the use of additional imaging modalities such as neuroinflammation,^45^ tau PET,^49^, ^50^, ^51^ advanced MRI techniques such as Neurite Orientation Dispersion and Density Imaging (NODDI),^52^, ^53^, ^54^ and other markers of synaptic density using [11C]‐UCBJ‐J PET.^55^ Further studies are warranted to investigate the metabolic and cognitive profiles associated with α‐syn pathology across the full spectrum of Lewy body diseases.^26^, ^56^ While the SAA+ and SAA− dichotomy is valuable for identifying the presence of pathological α‐syn, it currently lacks the granularity necessary to characterize nuances in disease progression and phenotypical trajectories as a function of α‐syn load. Further studies are needed to establish harmonized protocols for the quantitative analyses of α‐syn SAA based on its kinetic parameters (i.e., lag time and end‐point dilution).^12^, ^14^ Finally, while the Amprion SAA has been validated for the sensitive detection of pathological α‐syn in CSF, the reliance on CSF sampling may pose logistical challenges in larger clinical trials, because its perceived risk and slightly invasive nature may impact participant recruitment and retention. Future development of α‐syn assays from peripheral biofluids such as serum,^57^ oral mucosa,^58^ or saliva^59^ could expand accessibility in clinical research and enable more frequent longitudinal monitoring of disease progression.

## 5 CONCLUSION

As novel disease‐modifying therapies targeting α‐syn advance through the clinical trial pipelines,^60^, ^61^ our results highlight the potential value of incorporating FDG PET imaging and SAA biomarker status in patient selection and stratification. By identifying individuals with α‐syn–related hypometabolism, clinical trials in the AD spectrum may be better positioned to detect treatment effects and elucidate the mechanisms underlying the efficacy of these interventions. Furthermore, the mediation effects observed in our study suggest that FDG PET imaging could serve as a valuable outcome measure in clinical trials, providing a sensitive marker of treatment response and disease progression.

## CONFLICT OF INTEREST STATEMENT

E.M., S.A.P., H.J.W., and A.M.F. report no disclosures relevant to the manuscript. C.G.S. receives research support from the NIH. M.L.S. owns or has owned stock in medical‐related companies, unrelated to the current work, within the past 36 months: Align Technology, Inc., Inovio Pharmaceuticals, Inc., Mesa Laboratories, Inc., Johnson and Johnson, LHC Group, Inc., Natus Medical Inc., and Varex Imaging Corporation. C.R.J. Jr. is employed by Mayo Clinic. He receives grant funding from the National Institutes of Health (R37 AG011378, R01 AG041851), the Alexander family professorship, and the GHR Foundation. Within the past 36 months, he served on a DSMB for Roche pro bono; no payments to the individual or institution were involved. He has received funding from the Alzheimer's Association for travel to scientific meetings. R.C.P. serves as a consultant for Roche, Inc., Merck, Inc., Biogen, Inc., Eisai, Inc., Genentech, Inc., and Nestle, Inc.; served on a DSMB for Genentech; receives royalties from Oxford University Press and UpToDate; and receives NIH funding. B.F.B. has served as an investigator for clinical trials sponsored by Alector, Cognition Therapeutics, EIP Pharma, and Transposon. He serves on the scientific advisory board of the Tau Consortium, funded by the Rainwater Charitable Foundation. He receives research support from NIH, the Mayo Clinic Dorothy and H.T.M. Jr. Lewy Body Dementia Program, the Little Family Foundation, and the Ted Turner and Family Foundation. J.T.O. has no conflicts related to this study; unrelated to this work, he has received honoraria for work as DSMB chair or member for TauRx, Axon, and Eisai, has acted as a consultant for Roche, and has received research support from Alliance Medical and Merck. Dr. K.K. was partially funded by the Kathrine B. Andersen Professorship of Women's Health Research of the Mayo Clinic.  Dr. K.K. has served on data safety monitoring boards and/or was a Pfizer, Takeda, and Biogen consultant. She received research support from Eli Lilly. Author disclosures are available in the supporting information.

## CONSENT STATEMENT

The ADNI study was conducted in accordance with the Declaration of Helsinki, and the procedures were approved by the institutional review boards of all participating sites. All participants provided written informed consent at enrollment.

## Supporting information



Supporting information

Supporting information
