# Supplementary material for: Influence of alpha‐synuclein on glucose metabolism in Alzheimer's disease continuum: Analyses of α‐synuclein seed amplification assay and FDG‐PET
Source: Alzheimers Dement. 2025 Feb 12;21(2):e14571. doi: 10.1002/alz.14571 (PMC11815207; doi:10.1002/alz.14571)
Supplement: Supplementary file 1 — Supporting information [file ALZ-21-e14571-s001.docx]

## SUPPLEMENTARY MATERIAL

**
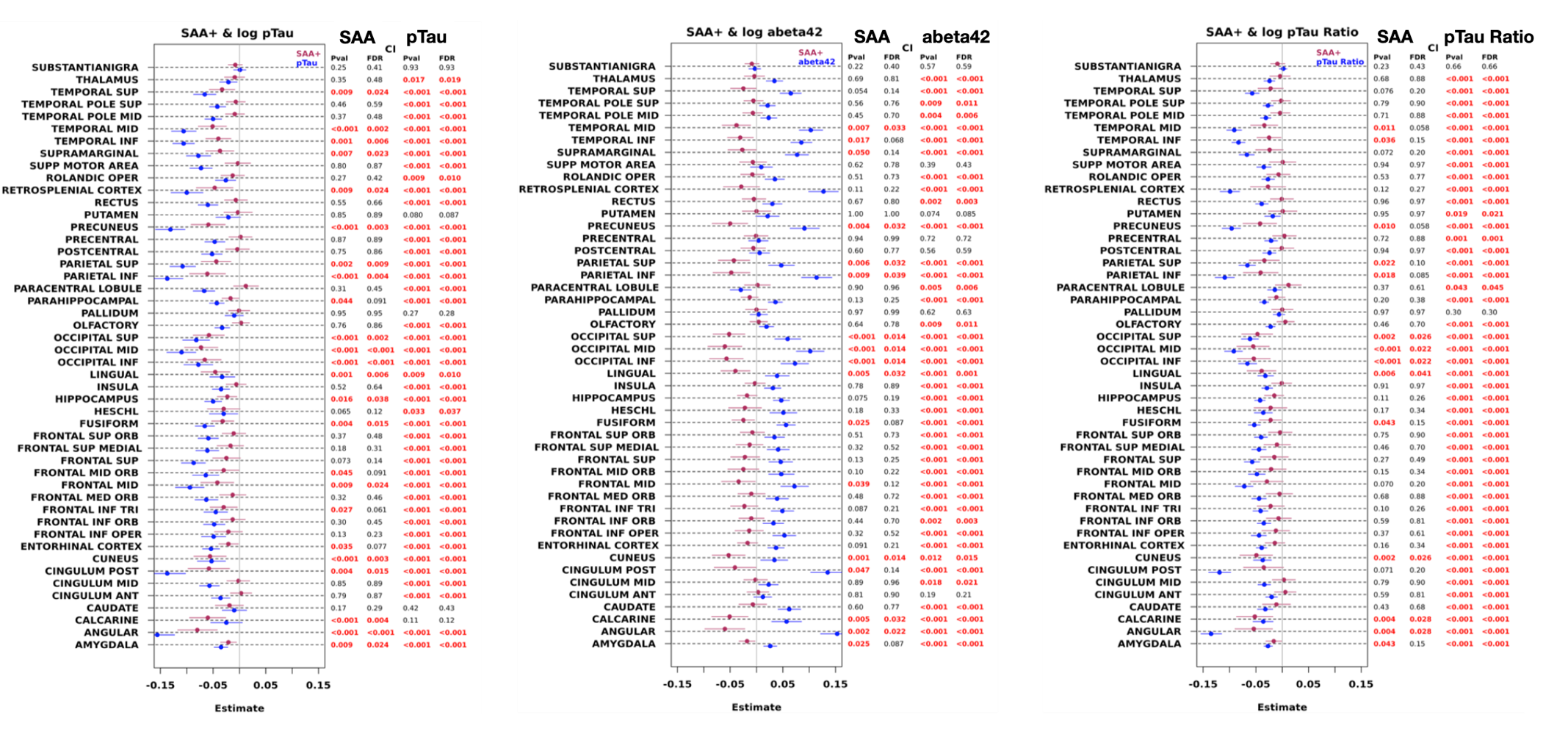
**

**Supplementary Figure 1. Forest plots illustrating regional associations between α-syn pathology (SAA+) and FDG-PET metabolism in cognitively impaired individuals while controlling for the CSF AD biomarker of the CSF p-tau181 / Aβ42 ratio.** The x-axis represents the effect estimate, with negative values indicating hypometabolism in α-syn SAA+ individuals. Abbreviations: α-syn, alpha-synuclein; Aβ42, amyloid-beta 42; AD, Alzheimer's disease; CSF, cerebrospinal fluid; FDG-PET, fluorodeoxyglucose positron emission tomography; p-tau, phosphorylated tau; SAA, seed amplification assay; SAA+, α-synuclein aggregates detected with an aggregation profile consistent with the characteristic seeding seen in Lewy body diseases


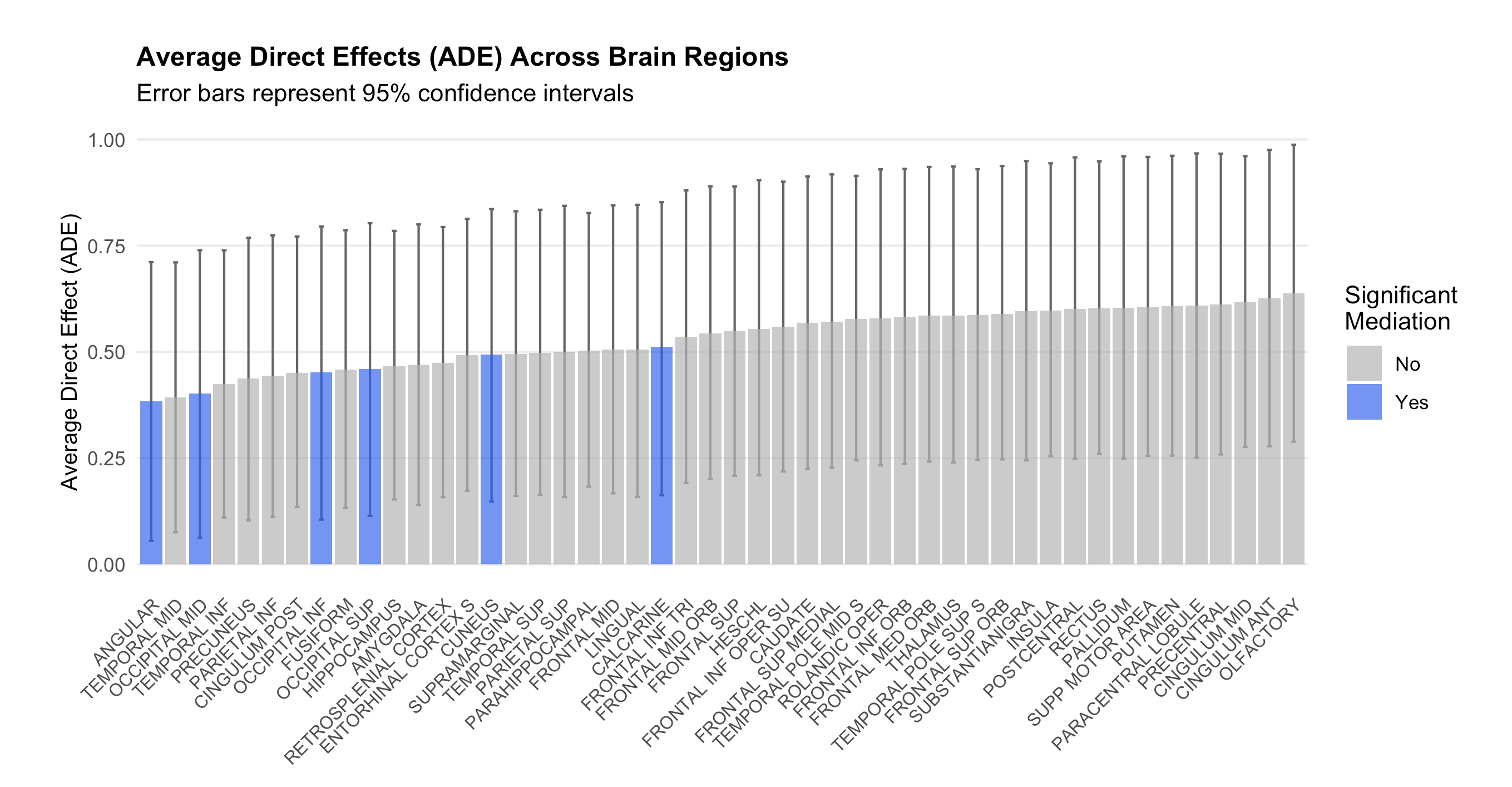


**Supplementary Figure 2. Average Direct Effects (ADE) of α-syn pathology on clinical severity across brain regions.** ADEs were derived from mediation analyses across brain regions, investigating how α-syn SAA directly influences clinical severity (CDR-SB) independent of its effects through brain metabolism measured using FDG-PET. Blue bars indicate regions that showed significant mediations (FDR q < 0.05) of regional FDG on the associations between α-syn SAA and CDR-SB. Error bars represent 95% confidence intervals. All regions showed significant direct effects (FDR q < 0.05), with ADEs ranging from 0.383 (Angular Gyrus) to 0.637 (Olfactory). All mediation analyses were adjusted for age, sex, site, APOE ε4 carrier status, and the CSF p-tau181/Aβ42 ratio. Abbreviations: ADE, Average Direct Effect; Aβ42, amyloid-beta 42; α-syn, alpha-synuclein; APOE, Apolipoprotein E; CDR-SB, Clinical Dementia Rating Sum of Boxes; CSF, cerebrospinal fluid; FDG-PET, fluorodeoxyglucose positron emission tomography; FDR, False Discovery Rate; p-tau, phosphorylated tau; SAA, seed amplification assay.
